# Initial clinical experience with total Müllerian compartment resection for locally advanced cervical cancer: a single-center preliminary retrospective cohort study and learning-curve analysis

**DOI:** 10.3389/fonc.2026.1865445

**Published:** 2026-07-24

**Authors:** Xinyou Wang, Jing Na, Yang Wang, Bingxin Han, Ya Li, Qiao Lu, Wenying Zhou, Yajie Duan, Jiawei Wang, Jiayuan Zhong, Shichao Han, Jun Wang

**Affiliations:** Department of Obstetrics and Gynecology, The Second Affiliated Hospital of Dalian Medical University, Dalian, China

**Keywords:** cervical cancer, membrane anatomy, membrane bridge, Müllerian compartment, radical hysterectomy

## Abstract

**Objective:**

In this study, we compared the clinical value of embryonic compartment hysterectomy—i.e., total Müllerian compartment resection (TMCR)—with traditional radical hysterectomy for treating locally advanced cervical cancer, and assessed the learning curve associated with this novel technique.

**Methods:**

We conducted a retrospective cohort analysis at the Second Affiliated Hospital of Dalian Medical University that comprised 60 women who underwent initial surgical treatment for cervical cancer (FIGO stages IB3 and IIA2) between May 2018 and May 2024. Participants were assigned to an embryonic-compartment hysterectomy group (n=37) and a traditional hysterectomy group (n=23). We collected demographic data, oncologic information, treatment-related complications, and follow-up data. Perioperative outcomes and oncologic results were compared between the groups, and the learning curve for TMCR was analyzed by applying cumulative sum (CUSUM) and best-fit curve analyses based on surgical duration.

**Results:**

We noted no significant differences in age, body mass index, tumor stage, pathologic type, surgical duration, or postoperative hospital stay between the two groups (P>0.05). However, the TMCR (“study”) group exhibited less intraoperative blood loss, with a median of 100 ml (50–200 ml) compared with 300 ml (110–500 ml) in the traditional group (P<0.05). Postoperative complication rates were similar, with 24.3% in the study group and 17.4% in the traditional group (P>0.05). The overall median follow-up duration was 30 months; however, the traditional group was followed for a median of 41 months compared with 23 months for the study group. All recurrences and deaths occurred in the traditional group. In addition, the median tumor size in the study group was 51 mm (IQR, 45–57.5 mm), compared with 46 mm (IQR, 42–50 mm) in the traditional group (P<0.05). The CUSUM curve peaked after the 12th case, indicating a transition from learning to proficient phases, with significantly reduced surgical times in the proficient phase (P<0.05).

**Conclusions:**

TMCR is safe and feasible for locally advanced cervical cancer. We posit that gynecologic oncologists will be able to achieve proficiency after performing 12 cases, with potential advantages in resecting larger tumors. However, further longitudinal studies and larger clinical trials are necessary to confirm these results.

## Introduction

Cervical cancer is a global health issue that seriously affects women (1–4). According to the World Health Organization (WHO), cervical cancer is the most common cancer among women in 23 countries and the leading cause of cancer-related deaths in 36 countries (5). Early-stage cervical cancer accounts for approximately 72.7% of cases, with a five-year survival rate of over 90% (6, 7). However, locally advanced cervical cancer (LACC) comprises 37% of cases, with a total five-year survival rate varying between 24.0% and 76.1% (8).

Despite recent advancements in treatment strategies, platinum-based concurrent chemoradiotherapy remains the standard treatment for many patients with LACC. However, treatment outcomes vary substantially according to tumor stage, tumor burden, lymph-node status, and treatment strategy, and recurrence remains an important clinical concern in selected patients (9). In addition, 23.3%–34.4% of patients experience tumor recurrence or metastasis after treatment (10). As a result, optimizing treatment strategies for selected patients with LACC remains a major challenge for gynecologic oncologists (11).

LACC can be categorized into broad and narrow definitions. The broader definition includes cervical cancer stages IB3 to IVA according to FIGO 2018 staging, while the narrower definition specifically includes stages IB3 and IIA2 (12). Both definitions are characterized by a local tumor diameter greater than 4 cm, without reaching the pelvic wall or distal vagina (13).

The larger size of local lesions in LACC leads to a higher incidence of intermediate-to-high-risk recurrence factors such as lymph node metastasis, parametrial invasion, and lymphovascular space involvement, and results in poorer prognosis. Radical hysterectomy is considered an alternative initial treatment option to concurrent chemoradiotherapy (14), a decision influenced by several factors. First, radiotherapy equipment is costly, and most advanced cervical cancer cases occur in economically underdeveloped countries (15, 16). Second, radiotherapy can cause irreversible ovarian dysfunction and vaginal epithelial fibrosis, significantly affecting a woman’s quality of life, especially given the rising incidence of cervical cancer among younger women (17). By contrast, complications from surgical intervention are often temporary and exert a relatively lesser impact on the quality of life and sexual health of young women compared with the permanent complications associated with chemoradiotherapy (18).

In patients with LACC, large tumor size and infiltrative growth patterns create indistinct boundaries with surrounding tissues, and this leads to significant vascular engorgement and increases the risk of intraoperative complications such as bleeding and organ injury. Intraoperative bleeding can obscure the surgical field, complicating complete tumor resection, and results in a higher rate of positive margins in traditional radical hysterectomy. This complexity increases the difficulty of the procedure and poses significant challenges for gynecologic oncologists, potentially deterring them from performing such surgeries. Therefore, enhancing the safety and feasibility of initial surgical treatments for patients with larger, locally advanced cervical cancer tumors has become a key area of research.

In the application of radical surgery for larger solid tumors, gastrointestinal surgeons were the first to conduct clinical trials on total mesorectal excision. Introduced by Heald et al. in 1982, this innovative surgical approach was based on fascial anatomy and utilized an avascular zone as the surgical entry point. This technique reduced the local recurrence rate of rectal cancer from 30%–40% to 5%–15%, significantly improving the prognosis for rectal cancer patients (19). Total mesorectal excision is now the gold standard for rectal cancer treatment. In gynecology, Höckel introduced the concept of total mesometrial excision in 2001, based on the embryonic development theory, and was the first to apply it to cervical cancer treatment (20).

This excision method achieved good oncologic outcomes in patients with early-stage, localized cervical cancer, and preliminary explorations were also conducted in patients with locally advanced tumors. Therefore, total mesometrial excision is promising in overcoming the limitations of traditional radical hysterectomy in the treatment of LACC. This technique applies a new anatomical understanding, employing the avascular zones between different embryonic origins as an entry point, and results in less bleeding and a reduced risk of organ damage while increasing the rate of negative surgical margins and decreasing the local recurrence rate. One particular study only comprised a small number of locally advanced cervical cancer cases, and although it was a prospective trial, it and most recent analyses were based on the FIGO 2009 staging, and, thus, there have been no reports under the new FIGO 2018 staging (12). These two staging systems reflect significant differences in defining locally advanced cervical cancer, and therefore new clinical trials are needed in the context of the revised staging system. In addition, some studies have shown that minimally invasive surgery exerts a notable negative impact on the oncologic outcomes of cervical cancer treatment (21). An investigation by Höckel and associates also included patients who underwent minimally invasive surgery, adding complexity to the interpretation of results (22). Therefore, in the current context, there were certain limitations regarding the reliability of these authors’ findings on cervical cancer outcomes.

Based on the theory of embryonic development, total Müllerian compartment resection (TMCR) has been preliminarily examined in patients with early-stage cervical cancer and exhibited favorable safety results (23). Our research team therefore combined the concepts of “membrane anatomy” and “Müllerian compartment” for LACC, following current international guidelines (FIGO 2018), and performed TMCR using an open surgical approach. We herein analyzed the quality-control data from a single center to compare the clinical value of TMCR with that of traditional radical hysterectomy. In addition, a cumulative sum (CUSUM) analysis was implemented to evaluate the learning curve for this technique, supporting the safety and feasibility of performing TMCR in patients with LACC.

## Methods

Using a retrospective, cohort-study approach, we enrolled 60 patients diagnosed with LACC (FIGO 2018 stages IB3 and IIA2) who were treated at the Second Affiliated Hospital of Dalian Medical University between May 2018 and May 2024. All women underwent surgical treatment: 37 underwent TMCR (the study group) and 23 underwent traditional radical hysterectomy (the traditional group). The surgeries in the study group were performed by the same experienced gynecologic oncologist.

Regarding inclusion criteria and according to the FIGO 2018 staging standards, all patients were diagnosed with cervical cancer stages IB3 or IIA2 and were between 18 and 75 years of age, with no prior history of chemotherapy or radiotherapy.

Our exclusion criteria encompassed patients with distant metastasis, other malignancies, prior abdominal surgeries, serious systemic diseases, mental health conditions, or cognitive impairment based on preoperative clinical evaluations.

We collected relevant patient data that encompassed age, BMI, histologic type, tumor grade, tumor size, surgical duration, intraoperative blood loss, hemoglobin changes during the perioperative period, specific blood loss and surgical duration for TMCR, intraoperative blood transfusions, length of hospital stay, postoperative complications, and outcomes of postoperative radiotherapy and chemotherapy. We also evaluated lymphovascular space invasion and lymph node metastasis in postoperative pathology and local and distant recurrence rates. Postoperative complications were graded using the Clavien-Dindo classification system (24).

This study involving human participants was reviewed and approved by the Ethics Committee of the Second Affiliated Hospital of Dalian Medical University (Approval No. KY2024-190-01). Informed consent was obtained from all participants either through written informed consent forms or through verbal consent by telephone during follow-up, as documented in the study records.

### Operative method

#### Traditional group

Surgical resection followed the Piver type III or QM C2 guidelines recommended by the NCCN, (25) focusing on organ removal (including the uterus and surrounding tissues). This approach used a combination of blunt and sharp dissection techniques, with ligament removal performed through separation, clamping, transection, and suturing. The main lateral pelvic ligament was cut horizontally inside the internal iliac vessels, whereas the posterior ligaments were removed near the sacral region. The ureter was freed for the anterior ligaments, and the vesicocervical and vesicovaginal ligaments were cut near the bladder. The cut ends were then clamped, transected, and sutured. Vaginal resection was performed at least 3 cm from the lesion.

#### Study group

The organs derived from the Müllerian embryonic primordium, together with their associated supporting structures, including nerves, blood vessels, ligaments, lymphatics, and other connective tissues, constitute a relatively integrated anatomical and functional system, which we define as the “Müllerian embryonic compartment.” The term “membrane bridge” is used to intuitively describe the connective interface between the Müllerian embryonic compartment and adjacent embryonic compartments. Through systematic separation and en bloc resection of these three-dimensional membrane bridges, compartment-based hysterectomy can ultimately be achieved.

1. The lateral parametrium, an “envelope” exit of the Müllerian embryonic compartment, contains the uterine artery, superficial and deep uterine veins, and surrounding lymphatic adipose tissue.

During embryologic development, each embryonic primordium is surrounded by its own membranous boundary. At the same time, it must communicate with adjacent organs and even with the whole body through blood vessels, lymphatic vessels, and nerves, thereby maintaining material exchange, signal transmission, nutritional supply, metabolic drainage, and the integrity of its functional system. The sites where these nerves, vessels, and lymphatics traverse the membranous boundary can be regarded as the so-called “envelope outlets,” through which the embryonic compartment within the “envelope” communicates with adjacent embryonic compartments and the systemic circulation for nutritional supply, metabolic drainage, and functional signal transmission. Because the Müllerian embryonic compartment is circumferentially surrounded by adjacent embryonic compartments, multiple envelope outlets exist around it. Among them, the most important lateral outlet corresponds anatomically to the lateral parametrium, which contains the uterine artery, superficial uterine vein, deep uterine vein, and surrounding lymphatic and neural tissues.

The lateral pelvic peritoneum overlying the round ligament, infundibulopelvic ligament, and iliopsoas muscle was incised to enter the lateral pelvic retroperitoneal space.

The surgical approach involves the use of the lateral retroperitoneal space to isolate the paravesical and Latzko’s pararectal spaces. From a membrane anatomy perspective, the paravesical space separates the posterior of the bladder from the envelope exit of the Müllerian compartment, while Latzko’s pararectal space separates the ureter from the same exit. These natural avascular spaces lie between the different embryonic compartments. The uterine artery and veins were then precisely clamped and transected to complete the lateral parametrium procedure.

2. The dorsal parametrium, made up of uterosacral ligaments, anchors the Müllerian embryonic compartment within the pelvis. From a membrane anatomy perspective, it involves separating the uterosacral ligaments and posterior vaginal wall of the Müllerian compartment from the hindgut compartment, which surgically corresponds to the rectovaginal space. This straightforward process requires the separation of only two embryonic compartments. By transecting the rectovaginal fold, the membrane bridge between these compartments, we accessed the avascular space between them, achieving a bloodless separation.
3. Ventral parametrium: The ventral parametrium area, due to the involvement of connective tissue between the parametrium and paracolpium of the Müllerian compartment, ureter of the ureteric bud embryonic compartment, and bladder of the urogenital compartment, is relatively challenging.

The membrane bridge between the Müllerian embryonic compartment and the urogenital sinus embryonic compartment is divided by the passage of the ureteric bud embryonic compartment into two parts: the cranial vesicocervical ligament and the caudal vesicovaginal ligament.

First, by dividing the membrane bridge between the Müllerian and urogenital compartments (vesicouterine fold), the avascular space between them is exposed, specifically the vesicocervical and vesicovaginal spaces. Expanding the vesicovaginal space laterally reveals the fourth space, further exposing another membrane bridge (vesicocervical ligament) between the Müllerian and urogenital compartments. After dividing this bridge, the connection between the ureteric bud and the Müllerian compartments became visible.

After transection of the vesicocervical ligament, the ureter can still be observed to remain closely attached to the parametrial tissue. Along the course of the ureteric bud embryonic compartment, a membrane bridge persists between the ureteric bud embryonic compartment and the Müllerian embryonic compartment. This connection, namely the membrane bridge, is divided in the depth of the “fourth space,” allowing gradual mobilization of the ureter and separation of these two embryonic compartments, thereby providing access to the paravaginal space.

Further dissection through the paravaginal space revealed the last membrane bridge (vesicovaginal ligament) between the Müllerian and urogenital compartments. Once this ligament is divided, the Müllerian compartment is completely isolated and positioned vertically toward the vagina. Cutting the paravaginal connective tissue at the levator ani muscle fascia completes this process.

**Figure 1:**
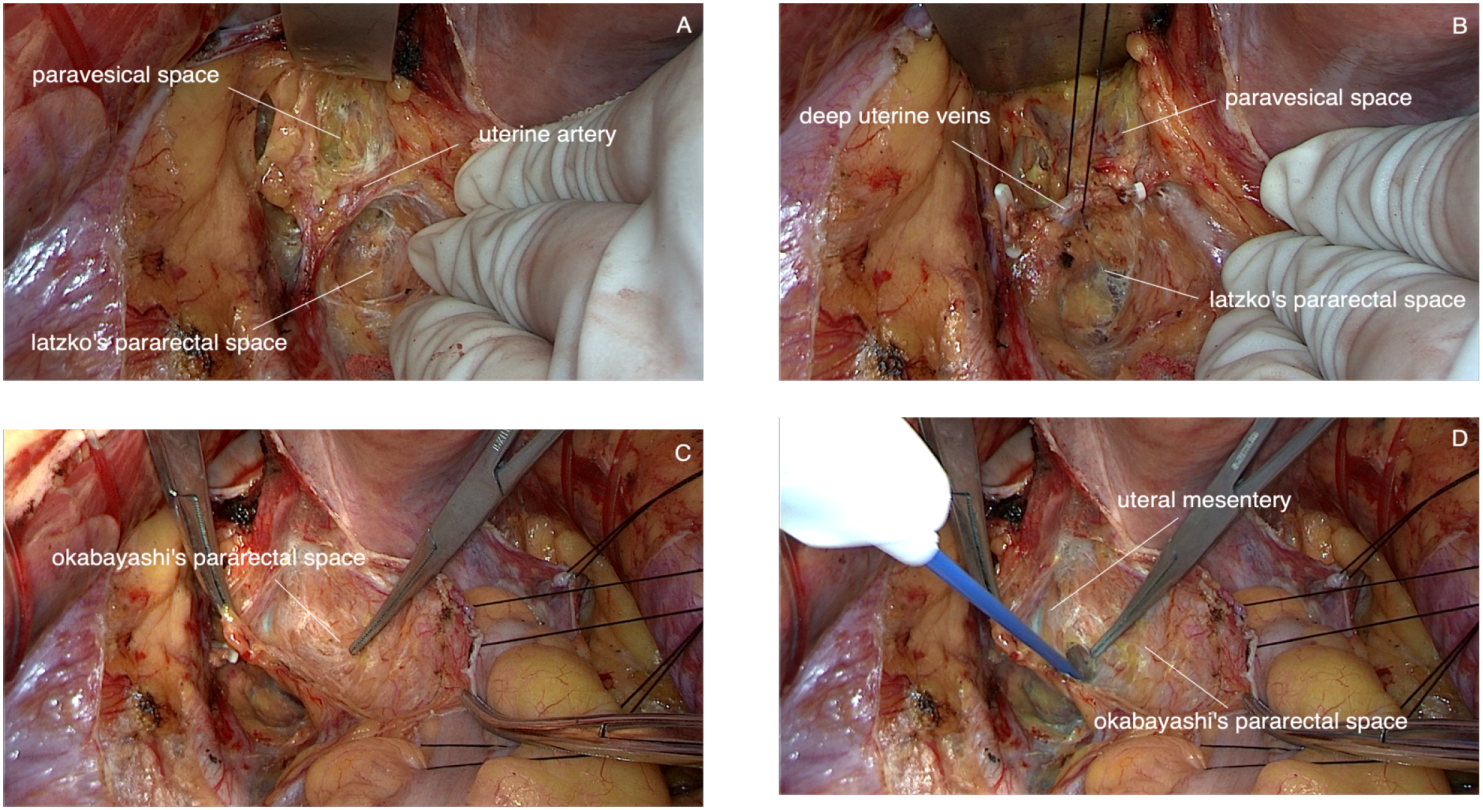
Dealing with the lateral parametrium. **(A)** Open paravesical space and latzko’s pararectal space to reveal uterine artery. **(B)** Transect uterine artery to reveal deep uterine viens. **(C, D)** Seperate ureter and its mesentery from rectal mesentery, open Okabayashi’s pararectal space.

**Figure 2:**
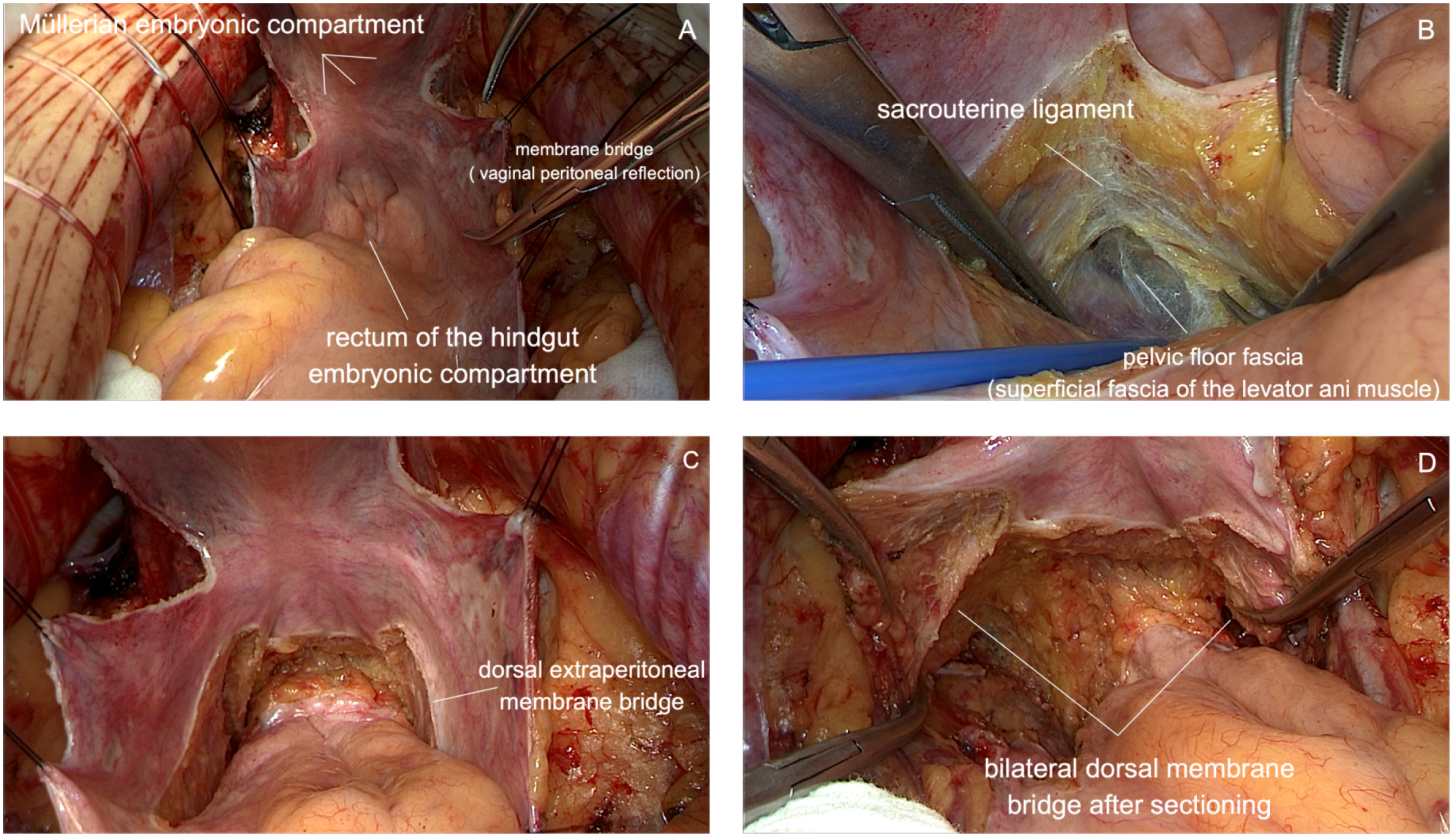
Dealing with the dorsal parametrium. **(A)** Membrane bridge at vesicouterine peritoneal fold. **(B)** Dissection of sacrouterine ligament. **(C, D)** Seperate the Müllerian embryonic compartment from the hindgut embryonic compartment and reveal bilateral dorsal membrane bridge.

**Figure 3:**
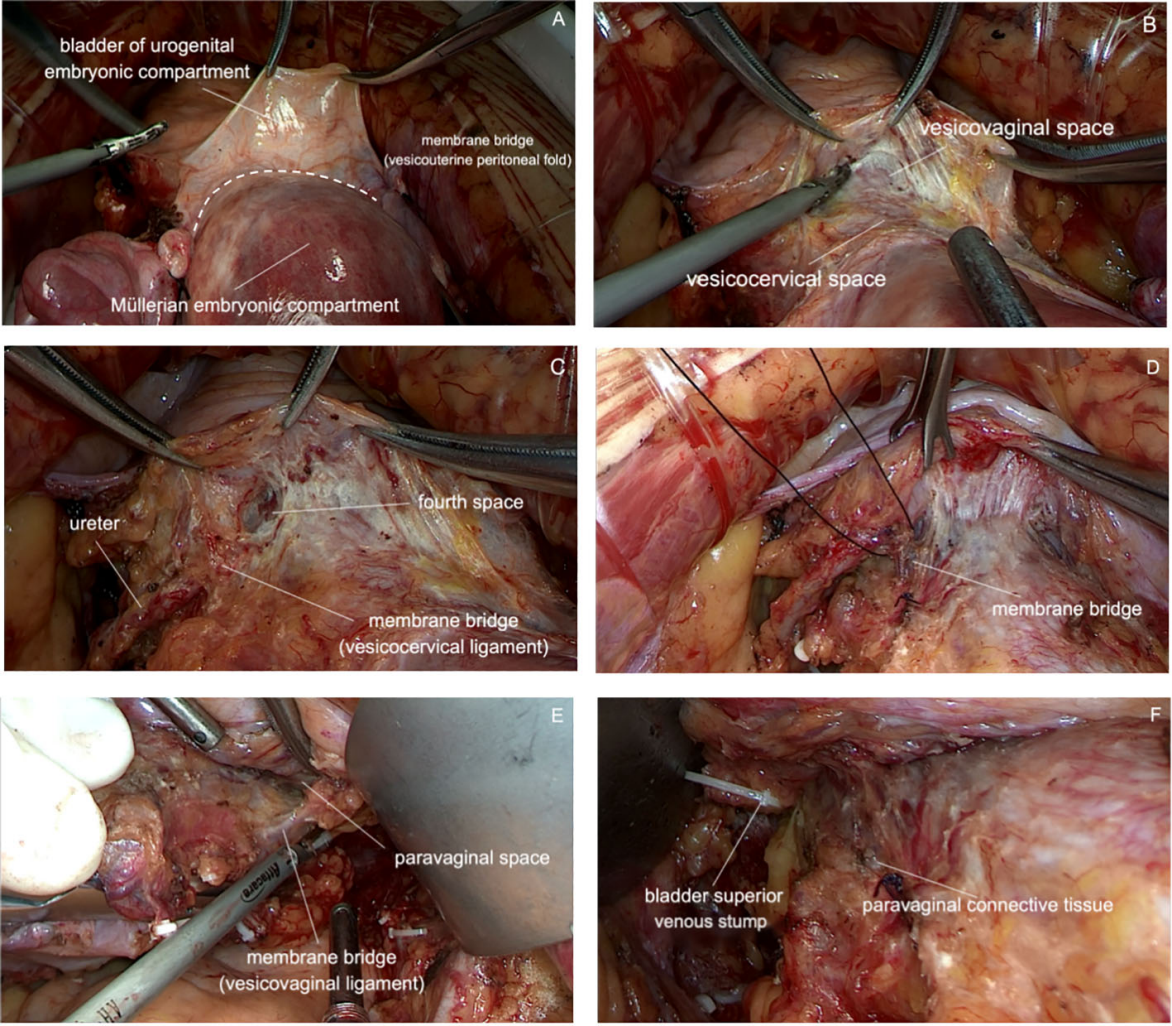
Dealing with the ventral parametrium. **(A)** Membrane bridge at vaginal peritoneal reflection. **(B)** Open vesicocervical and vesicovaginal space. **(C–E)** Membrane bridge at vesicocervical and vesicovaginal ligament. **(F)** Resection of paravaginal connective tissue.

**Figure 4:**
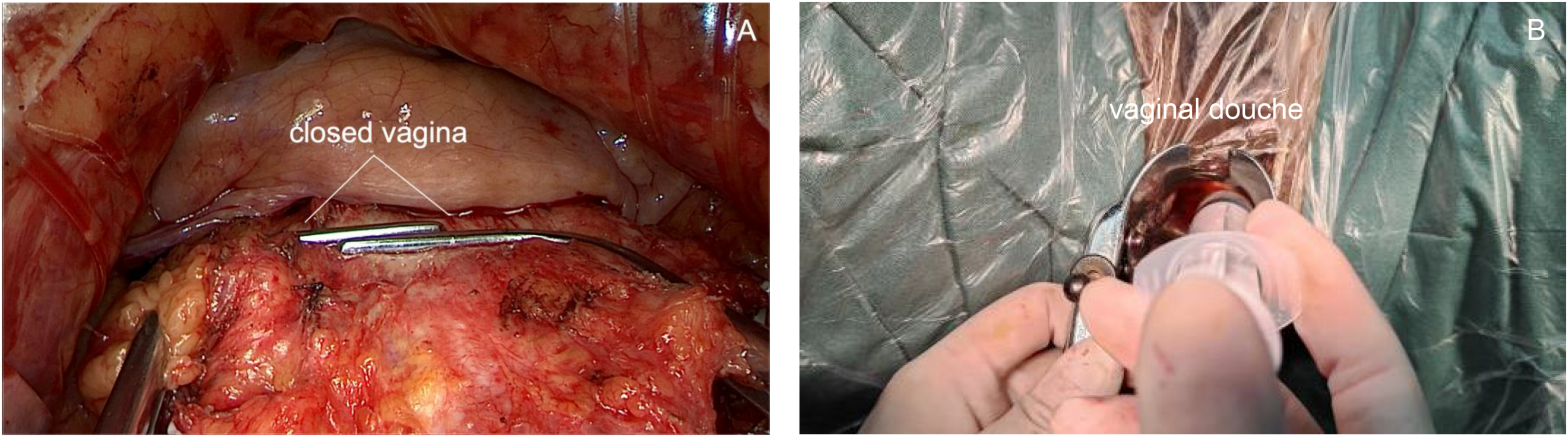
Tumor-free principle. **(A)** Close the vagina with forceps. **(B)** Douche the vagina.

4. Adhering to the tumor-free principle, two right-angle forceps were used to close the vagina. An assistant flushed the vaginal stump with 200 ml of sterile distilled water at 42 °C through the vaginal opening to remove any tumor tissue or cells from the vaginal wall before dividing the vagina. (**Figures 1**–**4**).

All patients underwent pelvic lymphadenectomy, and some patients selectively underwent para-aortic lymphadenectomy. Postoperative adjuvant treatment was recommended according to postoperative pathological risk factors, the NCCN guidelines, and multidisciplinary team evaluation. The treatment modalities included radiotherapy, chemotherapy, or concurrent chemoradiotherapy. High-risk factors included lymph-node metastasis, parametrial involvement, and positive surgical margins, whereas intermediate-risk factors included lymphovascular space invasion, large tumor size, and deep stromal invasion. Patients with high-risk factors were generally recommended to receive concurrent chemoradiotherapy, while those with intermediate-risk factors received adjuvant radiotherapy and/or chemotherapy according to clinical judgment.

All patients underwent perioperative venous thromboembolism risk assessment. Mechanical prophylaxis was routinely applied perioperatively. Prophylactic subcutaneous low-molecular-weight heparin was administered postoperatively according to institutional protocol when there was no contraindication related to bleeding risk.

### Statistical analysis

Data were statistically analyzed using SPSS 27.0. Normally distributed continuous variables are presented as means (standard deviation), with independent sample t-tests adopted for inter-group comparisons. Measurement data with skewed distribution were expressed as median (interquartile range, IQR), and the Mann–Whitney U test was implemented for inter-group comparisons. Categorical data are expressed as counts (percentages), and comparisons between groups were conducted using Chi-squared or Fisher’s exact-probability tests. We considered differences to be statistically significant at p < 0.05 (95% confidence interval) and calculated relative risk (RR) values.

All patients were assigned according to the sequence of surgeries, and the following formula was used for calculation: CUSUM = Σn i = 1(Ti-T), where Ti represented the surgical time for each patient undergoing the hysterectomy, T was the average surgical time, and n was the patient number.

The learning curve was plotted with the number of surgeries on the abscissa and the CUSUM values on the ordinate, using R software to fit the CUSUM learning curve. A P value of ≤0.05 determined successful curve fitting, and the model with the highest R2 value was selected as exhibiting the best fit. The vertex of the CUSUM learning curve was used to differentiate between the learning phase (up to and including the vertex) and the proficient phase (after the vertex). The x-coordinate value at the curve’s vertex indicated the minimal number of cumulative surgeries needed to surpass the learning curve.

## Results

There were no statistically significant differences (P > 0.05) in baseline data between the study group and the traditional group with respect to age, BMI, preoperative staging, pathologic type, or tumor differentiation (**Table 1**).

**Table 1:** The basic clinical data of the two groups of patients.

Patient characteristics	TMCR	RH	P	RR (95% CI)
Variable	(n=37)	(n=23)		
Mean age, SD	54.1 (12.3)	53.6 (11.2)	0.871	n.a
Mean body mass index, SD	23.8 (4.1)	22.2 (3.8)	0.141	n.a
Grade of differentiation				
G1-G2	25 (67.6)	10 (43.5)	0.158	n.a
G3	11 (29.7)	11 (47.8)		
Unknown	1 (2.7)	2 (8.7)		
Pathologic FIGO stage				
IB3	30 (81.1%)	17 (73.9%)	0.535	0.724 (0.278-1.886)
IIA2	7 (18.9%)	6 (26.1%)		
Tumor histology				
Squamous	36 (97.3%)	21 (91.3%)	0.290	0.216 (0.024-1.971)
Other	1 (2.7%)	2 (8.7%)		
Median tumor size, mm (IQR)	51 (45-57.5)	46 (42-50)	0.027	n.a
Lymph-vascular involvement;				
L0	9 (24.3)	7 (30.4)	0.868	n.a
L1	26 (70.3)	15 (65.2)		
Uncertain	2 (5.4)	1 (4.3)		
Lymph-node metastasis				
Yes	16 (43.2)	7 (30.4)	0.321	1.420 (0.697-2.894)
No	21 (56.8)	16 (69.6)		
Postoperative adjuvant therapy				
Yes	28 (75.7)	18 (78.3)	0.818	0.967 (0.727-1.287)
No	9 (24.3)	5 (21.7)		

TMCR, total Müllerian compartment resection;RH, radical hysterectomy;SD, Standard Deviation; IQR, Interquartile Range;n.a, not applicable; RR, relative risk.

### Intraoperative and postoperative comparisons

#### Intraoperative conditions

**Figure 5:**
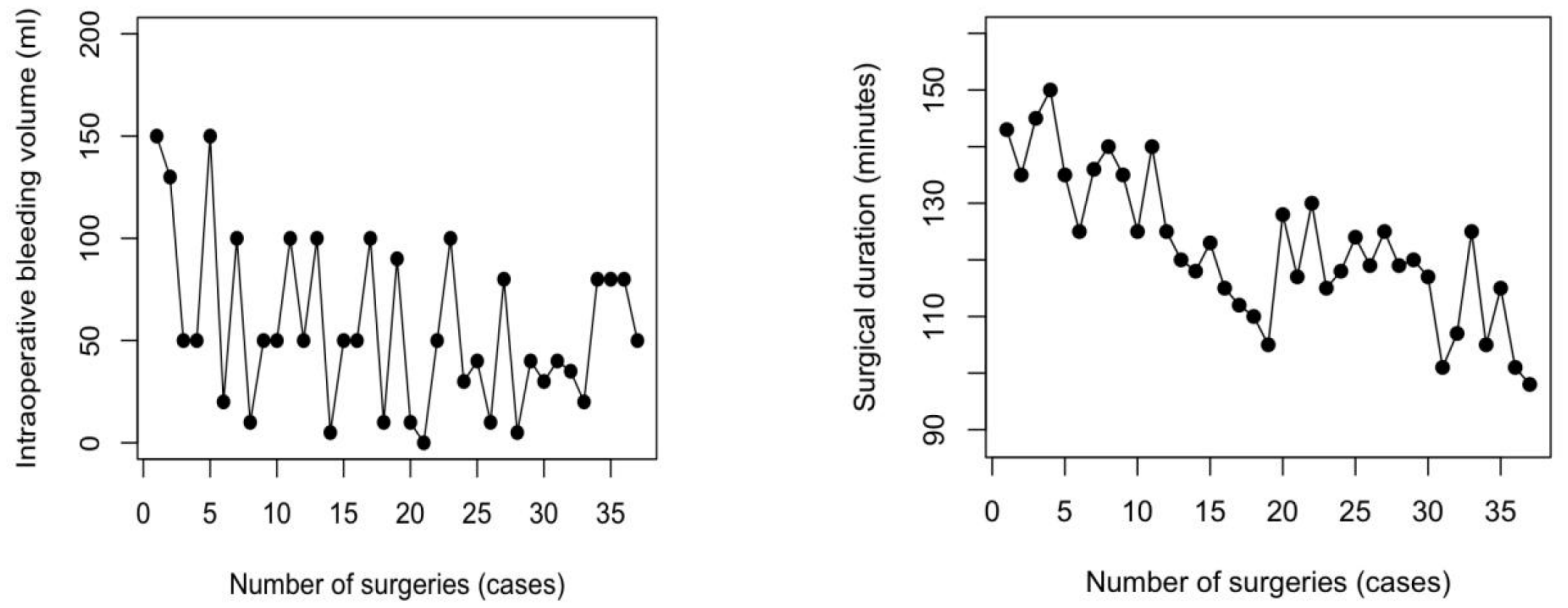
Intraoperative blood loss and surgical duration.

The two groups did not differ in terms of surgical duration, changes in perioperative hemoglobin levels, length of hospital stay, or incidence of postoperative complications. However, the study group exhibited significantly lower blood loss during surgery, with a median loss of 100 ml (IQR, 50–200 ml) compared with 300 ml (IQR, 110–500 ml) in the traditional group (p < 0.05) (**Table 2**). No patients in the study group required blood transfusions during or after surgery, while in the traditional group, one patient experienced a 700-ml intraoperative blood loss and received five units of leukoreduced red blood cells and 800 ml of plasma. For the study group, we recorded the blood loss and surgical duration specific to the TMCR group (i.e., the time from the opening of the retroperitoneal space to suturing the vaginal cuff), showing a decreasing tendency in both intraoperative blood loss and surgical duration as the number of cases increased (**Figure 5**).

**Table 2:** The basic surgical conditions of the two groups of patients.

Patient characteristics	TMCR	RH	P	RR(95% CI)
Variable	(n=37)	(n=23)		
Median length of surgery, min (IQR)	255 (234-307.5)	290 (240-310)	0.362	n.a
Median intraoperative median blood loss, mL (IQR)	100 (50-200)	300 (110-500)	0.002	n.a
Median length of hospitalization, days (IQR)	5 (5-7)	5 (4-8)	0.811	n.a
Median hemoglobin change value,g/L (IQR)	16 (6.5-23)	17 (9-27)	0.342	n.a
Postoperative complications				
Yes	9 (24.3)	4 (17.4)	0.526	1.399(0.490-3.995)
No	28 (75.7)	19 (82.6)		

TMCR, total Müllerian compartment resection;RH, radical hysterectomy;IQR, Interquartile Range;n.a, not applicable; RR, relative risk.

The median surgical duration (i.e., operative time from skin incision to completion of skin closure) for the study group was 255 min (IQR, 234-307.5min), while the traditional group exhibited a median duration of 290 min (IQR, 240–310 min), with no statistically significant difference between the groups. Our center recorded the time from the opening of the retroperitoneal space to suturing the vaginal cuff for the first time in the study group, with median procedural duration of 120 min (IQR, 115–132.5 min). Neither group experienced direct injuries to the urinary tract or intestines, or any other intraoperative complications.

#### CUSUM learning curve and clinical analysis

**Figure 6:**
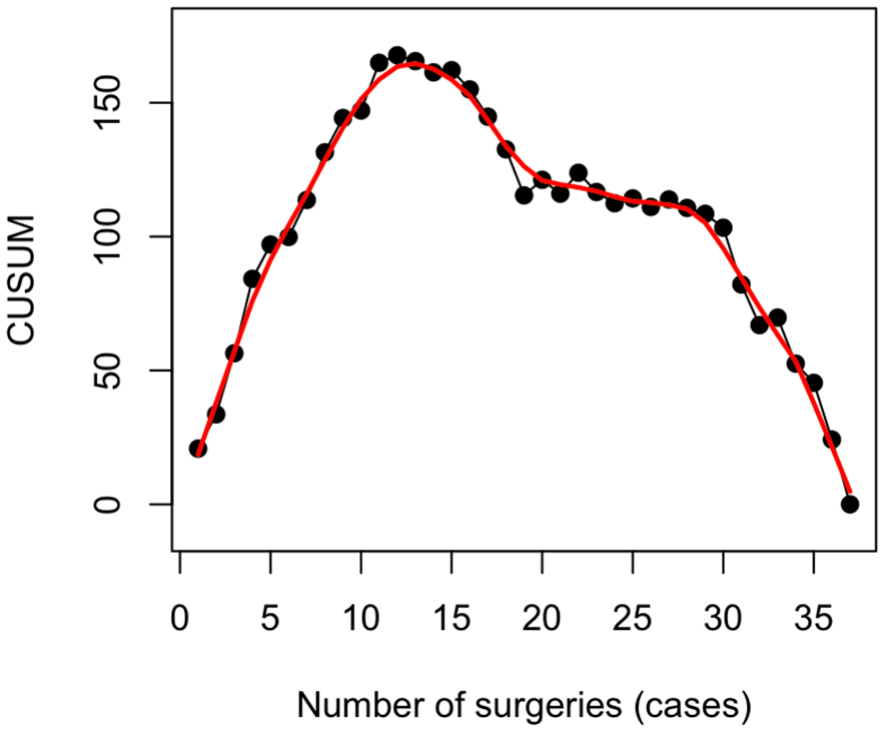
the CUSUM learning curve.

Using R software to fit the CUSUM learning curve, the best-fit equation was y=60.69x^3^−230.70x^2^−78.10x+105.16 (x represented the number of surgeries; the goodness-of-fit coefficient, R^2^ = 0.9002; P < 0.05) (**Figure 6**). The learning curve reached its peak after accumulating 12 cases, indicating that 12 surgeries were required to master the open hysterectomy procedure for patients with LACC. Based on the peak of the CUSUM learning curve, we divided the curve into two phases: the learning phase (1–12 cases) and the proficient phase (13−37 cases). A comparative analysis of the general conditions and perioperative indicators of patients in the two phases did not reveal any statistically significant differences in age, BMI, maximal tumor diameter, intraoperative blood loss, or postoperative complications (P > 0.05) (**Table 3**).

**Table 3:** Comparison of general conditions and perioperative indicators between the two stages.

Patient characteristics	Learning period	Proficiency period	P
Variable	n=12	n=25	
Mean age, SD	49.2 (12.6)	56.4 (11.7)	0.093
Mean body mass index, SD	23.5 (3.54)	23.9 (4.35)	0.755
Median tumor size, mm (IQR)	53.5 (47.25-61)	50 (44.5-57)	0.435
Median TMCR-specific procedural duration, min (IQR)	135.5 (127.5-142.25)	117 (108.5-121.5)	<0.001
Median intraoperative median blood loss, mL (IQR)	50 (50-122.5)	40 (15-80)	0.057
Postoperative complications, n (%)			
Yes	4 (33.3)	5 (20)	0.783
No	8 (66.7)	20 (80)	

SD, Standard Deviation; IQR, Interquartile Range.

#### Postoperative conditions

There was no statistically significant difference in the incidence of postoperative complications between the two groups. Thirteen patients developed Clavien-Dindo (CD) grade 1–3 postoperative complications. The major complications included secondary anemia, urinary tract infection, incomplete intestinal obstruction, pulmonary embolism, lower extremity deep vein thrombosis, hypoalbuminemia and hypokalemia.

Secondary anemia was the most common postoperative complication, occurring in 11 patients overall. The incidence was 13.5% (5/37) in the TMCR group and 26.1% (6/23) in the traditional radical hysterectomy group. Hypoalbuminemia occurred in five patients overall, with an incidence of 2.7% (1/37) in the TMCR group and 17.4% (4/23) in the traditional radical hysterectomy group. All related complications in the cases improved after symptomatic treatment (**Table 4**).

**Table 4:** Surgical complications in the two groups of patients.

Symptom	TMCR			RH		
	Grade I	Grade II	Grade III	Grade I	Grade II	Grade III
Anemia	4	1	0	3	3	0
Intestinal obstruction	2	0	0	1	0	0
Thrombus	0	1	1	0	0	0
Urinary system	0	0	0	1	0	0
Hypoproteinemia	0	1	0	0	4	0
Hypokalemia	0	1	0	0	0	0

TMCR, total Müllerian compartment resection; RH, radical hysterectomy.

According to the pathology results, there was a statistical difference in the diameter of local tumor lesions between the two groups. The lesions removed in the study group were larger, with a median maximal diameter of 51 mm (IQR, 45-57.5 mm). In addition, when we compared the number of lymph nodes removed and the metastasis rates between the two groups, we noted no statistically significant differences, and all postoperative pathology reports for the study group indicated negative margins.

### Follow-up data

The overall median follow-up duration was 30 months (range, 11–83 months). The median follow-up duration was 41 months (range, 20–83 months) in the traditional radical hysterectomy group and 23 months (range, 11–39 months) in the TMCR group. A total of five recurrence and/or death patients were observed in this study, all of which occurred in the traditional radical hysterectomy group.

Specifically, the first patient developed local recurrence 13 months after surgery and had been followed for 72 months postoperatively at the time of the last follow-up. The second patient did not complete postoperative adjuvant chemoradiotherapy as recommended, developed distant metastasis 12 months after surgery, and died 48 months after surgery. The third patient developed distant metastasis 11 months after surgery and was lost to follow-up at 22 months postoperatively. The fourth patient developed local recurrence 13 months after surgery and died 32 months after surgery. The fifth patient developed local recurrence 13 months after surgery and died 18 months after surgery.

Follow-up status was further analyzed according to whether postoperative adjuvant treatment was received. In the TMCR group, the median follow-up duration was 15 months (range, 12–38 months) for the nine patients who did not receive postoperative adjuvant treatment and 24.5 months (range, 11–39 months) for the 28 patients who received postoperative adjuvant treatment. As of the last follow-up, no recurrence or death was observed in the TMCR group, regardless of whether postoperative adjuvant treatment was administered.

In the traditional radical hysterectomy group, the median follow-up duration was 59 months (range, 22–65 months) for the five patients who did not receive postoperative adjuvant treatment. Among them, one patient developed distant metastasis and was lost to follow-up 22 months after surgery, while the remaining four patients had no recorded recurrence or death at the last follow-up. Among the 18 patients who received postoperative adjuvant treatment, the median follow-up duration was 41 months (range, 20–83 months). Four of these patients experienced recurrence and/or death, while the remaining 14 patients had no recorded recurrence or death at the last follow-up.

## Discussion

The principal goal of initial surgical treatment for patients with LACC is to remove the primary lesions of the cervix along with the surrounding tissues that are affected or potentially affected, including pericervical and vaginal tissues. Such a procedure will reduce local recurrence and distant metastasis, ultimately improving patient prognosis. Li’s team indicated that the chief drawback of directly performing traditional radical hysterectomy was the high rate of positive surgical margins post-operation, which also increased the likelihood of requiring adjuvant chemoradiotherapy (26). The incidence rates of complications such as lymphocysts, urinary tract injuries, and gastrointestinal dysfunction also rise significantly. Therefore, lowering the rate of positive surgical margins—thus minimizing perioperative complications and enhancing surgical safety—has become a topic of intense interest in gynecologic oncology. The introduction of aspects of mesenteric anatomy from gastrointestinal surgery has led to a novel surgical approach for rectal cancer termed total mesorectal excision and has significantly improved tumor prognosis. Likewise, it is essential in the field of gynecology to explore new surgical methods guided by the concept of mesenteric anatomy. Thus, in the present study and under the guidance of mesenteric anatomy and referencing Professor Höckel’s total mesometrial resection (TMMR) technique, we conducted a preliminary exploration of TMCR.

We acknowledged that intraoperative blood loss constituted an important indicator of surgical safety and feasibility during the examination of this novel surgical technique. Li et al. reported a median intraoperative blood loss of 400 ml (IQR, 200–600 ml) for patients with LACC undergoing traditional radical hysterectomy (27), while in our study, the median intraoperative blood loss in the traditional group was 300 ml (IQR, 110–500 ml). However, the median intraoperative blood loss in the study group was only 100 ml (IQR, 50–200 ml), showing a significant advantage over both our traditional group and those reported in previous studies. In a study by Vizzielli et al. where minimally invasive TMMR was used, the median intraoperative blood loss was reported as 150 ml (range, 50–200 ml) for the robotic group and 200 ml (range, 25–900 ml) for the laparoscopic group (28). Since we exclusively used an open surgical method in our study group, the intraoperative blood loss was even less than that noted with minimally invasive techniques. Moreover, a majority of patients in the Vizzielli study were early-stage cervical cancer patients based on the FIGO 2009 classification, with some undergoing preliminary corrections according to the FIGO 2018 guidelines (28). This implied that most of the tumors in these authors’ patients were smaller on average compared with those in our study, lowering the surgical difficulty in their cases. However, we achieved superior results with less blood loss, further supporting the safety of our TMCR procedure. These results indicated that compared with traditional anatomical concepts, the introduction of membrane anatomy clearly reduced intraoperative blood loss. The TMCR surgical approach that entails knowledge of the avascular spaces between different embryonic compartments therefore achieves significant advantages in controlling intraoperative bleeding.

The TMMR technique, combined with the QM classification for resection guidance, integrates the developmental concepts of the Müllerian duct’s embryonic compartments. By dissecting within the mesoscopic spaces of these embryonic compartments, we can clearly identify the anatomical landmarks for radical hysterectomy type QM-C, and this allows for the complete removal of the Müllerian duct’s embryonic compartment while achieving an ideal surgical view without compromising the extent of resection.

Performing surgical dissection based on the natural developmental boundaries gleaned from embryology allows for more precise separation and minimizes the risks of damaging major blood vessels. By contrast, traditional surgical methods can stretch and tear tissues, leading to unexpected bleeding. The embryonic compartment-resection technique reduces this risk through its precise dissection strategy, and when combined with modern minimally invasive techniques and hemostatic measures, it can effectively lower intraoperative blood loss and enhance the safety and success rate of the surgery.

A major concern for bleeding risk during extensive uterine resection is the ventral parametrial area, specifically the vesicocervical ligaments and the vesicovaginal ligaments. The ureters travel between the posterior bladder wall and the cervical-vaginal wall and are supplied by a web-like network of connecting blood vessels, making ventral parametrial resection quite challenging. To achieve the standard surgical resection boundaries, the risks of ureteral injury, bladder injury, or bleeding increase significantly.

From an embryologic perspective, recognizing the membranous boundaries between the ureteral buds’ embryonic compartment, the urinary embryonic compartment, and the Müllerian-duct embryonic compartments at the smooth chamber boundaries provides a complete and surgically accessible view. This makes it possible to fully separate each of the interconnected embryonic compartments. We ultimately attempted to completely excise the ventral parametrial tissue, guided by the QM-C resection range. The surgical team has thereby been able to preserve the membranous boundaries around the Müllerian duct’s embryonic compartment during our practical handling of the ventral parametrial area. Correctly identifying and separating the membranous spaces not only minimizes surgical bleeding but also optimizes the anatomy for the necessary transection of the uterine cervix and the vaginal wall; this then ensures the integrity of the embryonic compartment resection.

The surgical method used in the study group did not lead to a higher rate of complications after surgery. A total of nine patients experienced complications, generating a complication rate of 24.3%; of these, two cases involved pulmonary embolism. One of these patients was a 63-year-old woman with a BMI of 23.9 who was admitted with a hemoglobin level of 48 g/L. After receiving four units of leukocyte-reduced red blood cell suspension and two units of irradiated leukocyte-reduced red blood cells, her hemoglobin was corrected to 71 g/L before surgery. During the operation, total blood loss was 100 ml, and the tumor’s largest diameter was 67 mm. On the morning of the second post-operative day, the patient felt short of breath, and a bedside ultrasonogram revealed thrombosis in both external iliac veins and intermuscular venous thrombosis in both calves. A CT scan of the lungs revealed multiple emboli in the pulmonary arteries. The patient was then transferred to vascular surgery for the placement of a filter in the inferior vena cava and thrombus aspiration under local anesthesia, after which she gradually improved and was discharged.

The other patient with a pulmonary embolism was 52 years of age with a BMI of 26.6. She experienced 200 ml of blood loss during surgery and underwent pelvic lymphadenectomy and para-aortic lymph node dissection; the tumor’s largest diameter was 53 mm. On the third post-operative day, this patient developed shortness of breath after activity, and a lung CT scan showed multiple embolic changes in the pulmonary arteries. With assistance from the vascular surgery team and treatment with nadroparin, this patient improved and was discharged.

In addition, five patients developed anemia post-surgery, three of whom harbored pre-existing anemia. Blood loss during surgery was minimal; one patient had a pre-operative hemoglobin level of 95 g/L, which dropped to 71 g/L post-surgery, while another patient started at 134 g/L and then exhibited a level of 118 g/L post-surgery. One patient faced incomplete intestinal obstruction, and another exhibited incomplete obstruction with hypoalbuminemia and hypokalemia. We observed no intraoperative complications in our study group, which contrasted with the findings of Chiantera et al., who noted severe complication rates of 10%–47% during and after traditional radical hysterectomy (29–31). Urologic issues were the most common complications, with an injury rate of 1.45% and a functional impairment rate of 72% (32, 33). Urologic complications exert a significant impact on the post-operative quality of life for patients undergoing radical surgeries. In 2020, the European Society of Gynaecological Oncology (ESGO) included urological complications in their list of 15 quality indicators for cervical cancer surgery (33). In the present study, however, we recorded no instances of urologic complications. By following the principles of “membrane anatomy,” the embryonic compartment hysterectomy preserved most of the ureteral mesentery, which was congruent with research from Höckel’s team. By identifying the membranous gaps needed to retain the ureteral mesentery, we successfully avoided urologic complications (34). In the traditional group, we noted 4 cases of post-operative complications, resulting in a rate of 17.4%. Among these, one case involved urinary tract infection, hydronephrosis, and ureteral stricture; four cases involved anemia and hypoalbuminemia; and one case involved anemia alone. Although another patient faced intestinal obstruction and moderate anemia, all patients improved with symptomatic treatment. In a study by Ferrandina and co-workers, the complication rate for patients with LACC after undergoing traditional radical hysterectomy was reported to be 41.9%. Among these complications, urologic issues accounted for 38.9%, followed by vascular complications at 35.9% and gastrointestinal complications at 12.2% (35). In our study, the complication rate in the traditional group was 17.4%, chiefly due to post-operative anemia and hypoalbuminemia. The post-operative complication rate was 24.3% in our study group, but we observed no cases of urologic injury. Vizzielli and colleagues reported that using their minimally invasive TMMR surgical approach resulted in a 23.8% complication rate in the robotic group, with common issues of gastrointestinal adverse reactions and functional urologic disorders—each accounting for 14.2% (28). Their laparoscopic group exhibited a complication rate of 38%, mainly due to functional urologic disorders at 12.6%, with gastrointestinal adverse reactions at 3%. These findings indicate that relative to traditional radical hysterectomy, TMMR and TMCR show a significant advantage in controlling post-operative complications, particularly urologic issues.

Organ injuries are directly related to the scope of surgery and surgical techniques. In our study, the surgical scope involved the complete resection of the Müllerian-duct embryonic compartment—including the uterus and the associated blood vessels, lymphatics, adipose tissue, and fibrous connective tissues described in traditional anatomical surgeries. We adhered to the principles of preserving related autonomic nerves as much as possible and delicately dissected the connecting structures of the embryonic compartments (termed “membrane bridges”) to minimize extensive blunt dissection. This procedure was entirely performed without reducing the traditional range of radical hysterectomy. With clear visibility we successfully accomplished a complete resection of the embryonic compartment of the lateral mesonephric duct, ensuring the histologic integrity of tumor removal.

Reducing tumor recurrence and metastasis rates constitute the principal research goals when exploring novel surgical techniques, and these are directly linked to patient survival rates. There are data showing that early post-operative recurrences, especially centralized pelvic recurrences within the first year, are closely related to the surgical method employed (36). In traditional radical hysterectomy, the operation focuses on conventional anatomical landmarks for dissection, cutting, and ligation. This process inevitably causes bleeding or oozing from the surgical site, which can then lead to a lack of clarity in the operative field. Inadequate dissection of the paravaginal connective tissue and techniques that entail using large clamps for cutting and suturing can also increase the chances of leaving hidden or small residual lesions behind, especially in patients with LACC who manifest larger tumors. This situation then creates a heightened risk for post-operative recurrence.

In our analysis, the median follow-up duration was 41 months (range, 20–83 months) in the traditional radical hysterectomy group and 23 months (range, 11–39 months) in the TMCR group. Five patients experienced recurrence and/or death, all in the traditional radical hysterectomy group. These included three cases of local recurrence, two cases of distant metastasis, and three deaths. In the TMCR group, no local recurrence, distant recurrence, or death was observed during the current follow-up period. Nevertheless, because the TMCR group had a shorter follow-up duration and the number of events was limited, the absence of recurrence in this group should be interpreted cautiously as a short-term observation. Further long-term follow-up and larger prospective studies are needed to evaluate the oncologic efficacy of TMCR.

There are very few extant clinical studies on the initial use of TMMR surgery for LACC, and most authors have analyzed the method alongside surgery for local, early-stage cervical cancer. For example, the Höckel team enrolled 27 patients with LACC based on the FIGO 2009 staging system (37)—including both open and minimally invasive TMMR surgeries—and achieved a five-year disease-free survival rate of 67.4% (95% CI, 56.6%–76.1%), although they did not specify the timing of recurrences. There are even fewer reports on implementing traditional radical hysterectomy as the initial treatment for LACC.

While our study reflected a small number of cases, it is one of the first-ever reports to focus entirely on using open surgery to treat LACC in accordance with the FIGO 2018 staging and the LACC trial. Whether we compare them to a traditional surgery group or to Höckel’s team’s TMMR group, our results suggest encouraging short-term oncologic observations for locally advanced patients. This may have been due to the study group’s approach that was based upon membrane-dissection principles where surgeons typically used low-energy instruments for sharp dissection of the avascular spaces between different embryonic compartments. Through careful dissection, the surgeons avoided extensive blunt dissection that exposed the membrane bridges, allowing for precise cutting of the bridges. This method reduced the risk of clamping, cutting, and suturing large tissue masses, which in turn minimized the likelihood of leaving behind small residual lesions, and lowered the likelihood of recurrence.

We herein demonstrated that the median surgery time for the traditional group in this study was 290 min (IQR 240–310 min). Li and co-workers reported that performing traditional radical hysterectomy on patients with LACC took an average of 294.5 min for the open surgery group and 366.2 min for the laparoscopic group (38). By contrast, the median surgery time for embryonic compartment hysterectomy in the current study was 255 min (IQR 234-307.5min), as measured from the start of skin incision to completion of skin closure. This duration was similar to the surgical durations recorded by Vizzielli and colleagues for robotic and laparoscopic TMMR, which were 246 min (ranging from 180 to 300 min) and 260 min (ranging from 120 to 670 min), respectively (28). Unlike other studies, Our center recorded the time from the opening of the retroperitoneal space to suturing the vaginal cuff for the first time in the study group, with median procedural duration of 120 min (IQR, 115–132.5 min). Based on the “membrane anatomy” concept, this surgery exhibited minimal blood loss and a clear surgical field, further reducing surgical risk (39). This anatomical concept thus allowed for controlled surgical time while enhancing safety and effectiveness. These results indicated that for patients with LACC, embryonic compartment hysterectomy did not increase surgical time compared with traditional radical hysterectomy, thereby lowering the risk of post-operative complications.

Unlike the study by Höckel et al. that involved therapeutic lymphadenectomy (40), we herein opted not to perform therapeutic lymphadenectomy for the following reasons.

1. With respect to clinical guidelines (41), routine lymph node resection aligned with recommendations from authoritative guidelines such as the NCCN, and pathologic examination of the lymph nodes post-surgery provided critical staging information and supported subsequent adjuvant therapy.

2. As to challenges in tumor removal, when lymph node metastasis occurred and from an embryologic “membrane anatomy” perspective, surgery may have already exceeded the resection scope of the mesonephric duct embryonic compartment (22). Even with therapeutic lymphadenectomy, surgery limited to the embryonic compartment may not ensure complete tumor clearance and carries potential risks of distant metastasis, thus making post-operative adjuvant chemoradiotherapy crucial. Although research by Höckel’s team achieved favorable oncologic outcomes in early-stage cervical cancer cases, it did not show significant advantages in trial studies that involved locally advanced patients, particularly those who did not require chemoradiotherapy (37). This is one of the key reasons we did not choose therapeutic lymphadenectomy.

3. The focus of this study was to explore the impact of embryonic compartment hysterectomy on perioperative complications and short-term oncologic outcomes, particularly local recurrence. If therapeutic lymphadenectomy were performed simultaneously, it might have hindered the direct comparison with traditional surgical methods, making it difficult to determine the impact of different surgical modalities on final outcomes.

4. According to the current FIGO 2018 guidelines and for cases where preoperative imaging suggested lymph node positivity, lymph node staging surgery or concurrent chemoradiotherapy is recommended (42), rather than extensive hysterectomy. Therefore, such cases were not the focus of the present study. Höckel’s study (40) was based on FIGO 2009 criteria and did not encompass lymph node metastasis in staging; thus, it may have included more patients with lymph node metastasis, reducing data comparability.

5. With respect to surgical risk, the scope of therapeutic lymphadenectomy is broad, and the associated risks of bleeding and injury are significantly elevated. Therefore, performing such surgeries poses considerable challenges to even the most experienced gynecologic oncologists, potentially affecting their implementation in standard clinical practice.

With respect to the patients’ general information, our study revealed that although both groups fell under the FIGO 2018 staging of IB3 and IIA2 (i.e., harboring tumors greater than 4 cm in diameter), we refined our statistics to highlight differences in tumor sizes as the size of the local tumor was closely linked to surgical difficulty and tumor prognosis. We subsequently discerned significant differences in tumor sizes between the study group and the traditional surgery group. According to Vizzielli’s investigation, tumors up to 62 mm in diameter could be safely removed via laparoscopy (28). In our study group, the median tumor size was 51 mm (IQR, 45–57.5 mm) in the TMCR group and 46 mm (IQR, 42–50 mm) in the traditional radical hysterectomy group. Despite this, the membrane-anatomy surgery group achieved excellent blood-loss control and favorable short-term oncologic outcomes. This finding suggests that with extended follow-up time, embryonic compartment hysterectomy based on embryologic origin under the membrane-anatomy concept holds promise as a safe initial treatment option for patients with LACC.

In addition, unlike other studies, ours was the first-ever to separately record the blood loss specifically from the TMCR procedure, excluding any bleeding from lymph node removal. The mean intraoperative blood loss was 56 ml (ranging from 0 to 150 ml). While the number of surgeries was limited, the initial results suggested a clear tendency for decreasing blood loss as additional TMCR procedures were performed, indicating that enhanced surgical skills significantly contributed to improved control of blood loss during surgery (43, 44).

In our study, the CUSUM learning curve—fitted to the surgery duration—peaked after 12 accumulated cases, suggesting that for gynecologic oncologists familiar with traditional radical hysterectomy, 12 cases were needed to master embryonic compartment hysterectomy. Cases 1–12 constituted the learning phase, where the surgeon was familiarizing themselves with the procedure, hence the incline in the curve. Cases 13–37 represented the proficiency phase, where the surgeon had mastered the characteristics and techniques of the procedure, resulting in a decline in the curve.

There were no significant differences between the learning and proficiency phases in terms of age, BMI, tumor size, intraoperative blood loss, or surgical complications—indicating a safe and reliable learning process. The complication rate was 33% in the learning phase and 20% in the proficiency phase, with no difference between them. This suggests that the occurrence of complications in performing embryonic compartment hysterectomy for locally advanced patients may be more likely related to the severity of the disease itself rather than the surgeon’s familiarity with the procedure.

This study on the learning curve only represented the process of learning embryonic compartment hysterectomy under the premise of proficiency in traditional gynecologic surgery. Further large-sample, multicenter clinical trials are still needed to refine the learning curve. While this study was a single-center, retrospective cohort study, we preliminarily demonstrated that TMCR based on the membrane-anatomy concept showed promising perioperative outcomes and oncologic results for patients with LACC undergoing initial surgical treatment. Extending the follow-up period and collecting relevant data will facilitate a more thorough evaluation of the long-term efficacy and safety of the surgery and will pave the way for potential large-sample, multicenter, randomized controlled trials in the future.

This study on the learning curve only represented the process of learning embryonic compartment hysterectomy under the premise of proficiency in traditional gynecologic surgery. Further large-sample, multicenter clinical trials are still needed to refine the learning curve.

While this study was a single-center, retrospective cohort study, we preliminarily demonstrated that TMCR based on the membrane-anatomy concept showed promising perioperative outcomes and oncologic results for patients with LACC undergoing initial surgical treatment. Extending the follow-up period and collecting relevant data will facilitate a more thorough evaluation of the long-term efficacy and safety of the surgery and will pave the way for potential large-sample, multicenter, randomized controlled trials in the future.

## Conclusion

In summary, TMCR guided by the membrane-anatomy concept constitutes a safe and feasible treatment option for LACC, may facilitate anatomical resection of large local lesions in selected patients with LACC. For experienced gynecologic oncologists, the learning curve was short and the process was safe. This surgery not only reduced the risk of intraoperative bleeding but also showed encouraging short-term oncologic observations. We anticipate that long-term follow-up and multicenter clinical trials will further validate the safety and efficacy of TMCR (based on embryologic origins) in treating LACC.

## Data Availability

The raw data supporting the conclusions of this article will be made available by the authors, without undue reservation.
